# Nervous Diseases

**Published:** 1906-08-11

**Authors:** 


### Progress in Medicine and Surgery.

### NERVOUS DISEASES.

**Shoemaker's Cramp.**—Tetany forms the subject of a careful paper by C. P. Howard. The disease seems first to have been described in 1830 by Steinheim, of Altona, as a rare and severe form of rheumatism. Dance and his pupils and Trousseau have contributed largely to our knowledge of the subject. The disease is commoner in Europe than in America, perhaps owing to the greater frequency of one of its predisposing factors, rickets, on this continent, is most prevalent in winter and spring, and affects chiefly infants and young adults. Males seem more susceptible than females, and a family tendency is sometimes apparent. Adult cases seem to be more common in the working classes, especially among those following a sedentary occupation, such as shoemakers and tailors. In certain German towns it has been found that shoemakers furnished 43 per cent. of all the cases, although constituting only 1.3 per cent. of the population. Frankl-Hochwart has formulated an ætiological classification, and he describes an epidemic tetany or "shoemaker's cramp" especially affecting young men of the working classes. This form is acute, of short duration, rarely fatal, and possibly infectious. Association with gastro-intestinal disorders, such as diarrhœa, dyspepsia, gastrectasis, and intestinal worms, is frequent—60 to 80 per cent. Gastric tetany forms a characteristic group. The present writer reports four cases of his own associated with dilatation of the stomach. Hyperacidity and hypersecretion without obvious dilatation are also ætiological factors. Severe vomiting, lavage, or percussion over the stomach may precipitate an attack. The acute specific fevers, such as typhoid, measles, scarlet fever, cholera, and influenza; poisons such as chloroform, morphine, ergot, lead, and alcohol; and certain nervous disorders, such as intracranial tumours, syringomyelia, and exophthalmic goître form other predisposing groups. Tetany may also be associated with thyroid insufficiency and has followed the removal of the parathyroids. The theory of deficient thyroid activity has been brought forward also to explain the association of tetany with pregnancy and lactation, of which 20 or 30 cases have been recorded. Among children gastro-intestinal disorders, rickets, and acute infections are the chief predisposing factors. Laryngismus stridulus and carpo-pedal contractions are now regarded as manifestations of tetany. Nothing definite is known of the morbid anatomy of the disease. Hyperæmia of the cord and brain, perivascular inflammation of the spinal blood-vessels, peripheral neuritis, and inflammation of the anterior cornual cells have been described by various writers. In one of Howard's own cases MacCallum found a great number of karyokinetic figures in the parathyroid glands. The pathology is mere guesswork. Various theories have been advanced such as the dehydration theory of Kussmaul, who suggested a nervous instability and irritability consequent on a draining of the tissues of fluid in gastro-intestinal disorders, and a theory of reflex irritability due to hypersensitiveness of the gastric nerve endings.

**Auto-Intoxication.**—The theory of autogenous intoxication is more attractive and the stomach and the thyroid gland have been suggested as two possible sources of the formation of toxins. The characteristic symptom of the disease is the occurrence of muscular cramps which are liable to be induced by constitutional disturbance, over-exertion, or manipulations, and are preceded by definite prodromata, such as numbness, tingling and formication in the extremities, vomiting, and headache. The spasms, most commonly affecting the hand, and producing the position known as the "obstetric hand," are bilateral and tonic in character and usually commence at the periphery and gradually spread upwards. The feet assume the position of talipes equino-varus. Spasm of the facial muscles may produce the "risus sardonicus," and trismus, though rare, does occur in tetany as well as in tetanus; opisthotonus, laryngeal spasm especially in ricketty children, and, rarely, spasm of the diaphragm and bladder also occur. Different opinions are expressed by various observers regarding the relation of laryngospasm or laryngismus stridulus, to tetany. In some series of cases laryngismus and tetany appear to occur but rarely in the same patient. Most writers, however, now hold that the majority of cases of laryngismus are associated with tetany. There are certain accessory phenomena which are more or less characteristic of tetany, and therefore of assistance in diagnosis. Trousseau's phenomenon, said to be often present also in hysteria, is best marked in adults. It consists in the production of typical spasm by pressure on the nerve trunks and blood-vessels of the affected limb, especially of the arm. Chvostek's phenomenon implies increased mechanical irritability of the motor nerves and of the muscles. Spasm is produced by tapping the motor nerve supplying the affected muscles. Various facial contractions can be produced by tapping the face or the upper lip. Similar manifestations can also be obtained in epilepsy, hysteria, chorea, and other nervous complaints. Erb's phenomenon is very constant and persists for weeks after the disappearance of the spasm. It consists in an increased excitability to the faradic and especially to the galvanic current. Application of the cathode to the nerve has produced tetanus of the muscles supplied in proportion to the strength of the current.

**A New Sign.**—Peters has recently called attention to the "jumping-jack" sign. Application of the anode to the breast and the cathode to the lower cervical vertebræ causes the arms, on the passage of a current, to be jerked up and out like a child's toy. This observer has failed to obtain the sign after lumbar puncture and so assigns it to increased pressure of cerebro-spinal fluid. Loss of consciousness is rare during the spasms, which are sometimes intensely painful. Paræsthesiæ also occur, but anæsthesia is rare. Hoffman has observed an increased irritability of the sensory nerves. An irregular contraction of the left half of the diaphragm synchronous with the heart-beat has been described by Solovieff. Vaso-motor and trophic disturbances occur in chronic cases, such as flushing, cutaneous eruptions, falling of the hair, etc. Of general symptoms, the pulse-rate and breathing are often accelerated, and temperature may be moderately raised. The urine, as a rule, is diminished and may contain albumin. Tetany may sometimes be confused with hysteria or with Jacksonian epilepsy. In the latter, however, the paroxysms are unilateral at first and general convulsions are accompanied by loss of consciousness. Tetanic spasms sometimes occur in the tuberculous meningitis of children. In tetanus the spasms are more continuous and less intermittent, and beginning in the masseter muscles they spread towards the trunk and limbs, whereas in tetany the spasms spread from the periphery upwards towards the centre. Treatment must be directed as far as possible to removing the cause on which the spasms appear to depend. The latter may be partially relieved by rest, cold affusions, baths, hot packs, and the administration of sedatives such as opium, bromide, and chloral.

**Strychnine and Tetanus Poisoning.**—The treatment of strychnine and tetanus poisoning by the production of spinal anæsthesia has been advocated by A. E. Russell. His remarks are based on some experiments made by Professor Sherrington, who has demonstrated the rule that whenever a group of muscles contracts there is a corresponding inhibition of the opposing group of muscles, but that in strychnine and tetanus poisoning this reciprocal inhibition is converted into a violent excitation, so that when, for instance, the patient wishes to open his mouth, trismus, a violent action of the muscles closing the mouth, follows and the typical "lock-jaw" results. One is tempted to inquire, since this is so, whether temporary benefit might not result from persuading the patient to strongly attempt to do the opposite of what he would normally intend, *e.g.* to clench his teeth firmly when attempts at feeding are being carried out. L. Corning first described and suggested the use of spinal anæsthesia in the treatment of convulsions due to these poisons, and experimental observations on animals have shown that convulsions are at once checked by the injection of cocaine into the spinal subdural space. Eucaine $\beta$ is preferable to cocaine as it is less toxic and can be sterilised without decomposition. The dose required is very small, one or two cubic centimetres of a 3-per-cent. solution of the former or of a 1-per-cent. solution of cocaine, or smaller doses may be employed at first. By such an injection made by lumbar puncture complete analgesia of the body may be obtained for one or two hours, during which time the convulsions of strychnine, which is a rapidly eliminated poison, are in abeyance from the cutting off of afferent impressions. A case of tetanus poisoning in a boy was successfully treated by G. B. Murphy by successive injections of morphine, eucaine, and salt solution. Only $\frac{1}{17}$th of a grain of cocaine and $\frac{1}{75}$th of a grain of morphine were injected at once. Since the amount of spinal fluid aspirated was always considerably in excess of the amount of solution injected the relief of pressure may have had something to do with the favourable result.

